# Quantitative and qualitative analyses of grafted okra for corrosion inhibition of mild steel in acidic medium

**DOI:** 10.3389/fchem.2023.1129673

**Published:** 2023-02-24

**Authors:** Aliyu Adebayo Sulaimon, Pearl Isabellah Murungi, Bennet Nii Tackie-Otoo, Princess Christiana Nwankwo, Mohamad Azmi Bustam

**Affiliations:** ^1^ Department of Petroleum Engineering, Universiti Teknologi Petronas, Tronoh, Malaysia; ^2^ Centre of Research in Ionic Liquids (CORIL), Universiti Teknologi Petronas, Seri Iskandar, Malaysia; ^3^ Department of Petroleum Engineering, University of Ibadan, Ibadan, Nigeria; ^4^ Department of Chemical Engineering, Universiti Teknologi Petronas, Tronoh, Malaysia

**Keywords:** corrosion inhibitors, graft polymer, okra mucilage, box-behnken, analysis of variance, polyacrylamide

## Abstract

**Introduction:** Natural plant polymers demonstrate effective corrosion inhibition abilities, because of their numerous binding sites and excellent adsorption abilities.

**Methodology:** In this study, the Box-Behnken method, gravimetric and electrochemical analyses were used to design and investigate the corrosion inhibition potential of a modified graft polymer of okra for mild steel in a 1M HCl medium. The influence of inhibitor concentration, temperature, and time were also investigated. Qualitatively, the Fourier Transform Infrared (FTIR) spectroscopy, Thermogravimetric Analysis (TGA), and Field emission scanning electron microscopy (FESEM) were used to characterize the extracts and evaluate the metal’s surface morphology.

**Results and discussion:** The quantitative analyses showed that the modified natural polymer’s inhibition efficiency (IE) increased with concentration and reached 73.5% at 800 ppm, with a mixed-type mode of inhibition. From the response surface methodology, it was revealed that temperature influences the IE more than concentration and immersion time. The optimized IE using the desirability function showed the possibility of attaining 88.2% inhibition with inhibitor concentration at 142.3 ppm, temperature at 60.4°C, and an immersion time of 22.4 h. The new functional groups in the hybrid polymer revealed by FTIR analysis shows that grafting improved the inhibitor’s adsorption abilities. TGA analysis confirmed the extract’s high thermal stability, which highlights the inhibitor’s strong adsorption and efficiency for high temperatures. FESEM analysis indicated evidence of inhibitor adsorption onto the metal surface.

**Conclusion:** These findings suggest that the grafting of okra with acrylamide enhances its inhibition properties and contributes to its functionality as a cost-effective plant-based alternative inhibitor against corrosion for mild steel facilities.

## 1 Introduction

Corrosion is an irreversible surface reaction that occurs between a material and its surroundings, resulting in its deterioration (Dwivedi et al., 2021). The use of acid solutions in pickling procedures, chemical cleaning, and oil well acidification is a common occurrence that may result in a decline in metals’ integrity which could affect industrial systems, the environment, and human life (Lagrenee et al., 2001; Fayomi et al., 2019; Miralrio and Espinoza Vazquez, 2020; Zhang et al., 2020; Dwivedi et al., 2021; Murungi and Sulaimon, 2022). Corrosion inhibitors are the commonest form of corrosion remediation, especially for closed-system applications (Raja and Sethuraman, 2008; Bharatiya et al., 2019; Chaubey et al., 2020). Many of the conventional synthetic corrosion inhibitors have been found to be toxic, with compositions of chromium ions, formamide, arsenics, and other harmful substances, leading to their ban (Raja and Sethuraman, 2008; Umoren et al., 2008; Bahlakeh et al., 2019; Miralrio and Espinoza Vazquez, 2020). New frontiers in eco-friendly corrosion inhibitors reveal that natural polymers are highly efficient alternatives to synthetic inhibitors. They have versatile properties such as large numbers of binding sites in a single molecule, effective anchoring properties due to several electron-rich sites, and O_2_ and N_2_ heteroatoms in their structure that interact with unpaired electrons on metals to enhance adsorption (Banerjee et al., 2012; Fathima Sabirneeza et al., 2015; Azzam et al., 2018). Some plant-based natural polymers with excellent surface adsorption abilities have shown an increase in metal corrosion inhibition with their increasing concentration. These results may be attributed to the abundance of -OH, COOH, and -NH_2_ functional groups in their structure (Babaladimath et al., 2018; Quy et al., 2019). These extracts are commonly composed of different bioactive components such as alkaloids, polyphenols, tannins, steroids, terpenoids, flavonoids, phenols, saponins, and glycosides (Verma et al., 2018; Heidarshenas et al., 2020). Extracts with high-water holding capacity have also been attributable to the presence of numerous hydroxyl groups and protein molecules in their structures (Tosif et al., 2021). The mucilage of Plantago ovata (Mobin and Rizvi, 2017) was studied for its corrosion inhibition ability for carbon steel, in 1M hydrochloric acid (HCl), and an, I.E of 93.5% was observed with 1000ppm of the extract. Aloe polysaccharide extract (Zhang et al., 2020) was also investigated for corrosion inhibition abilities, where 800 mg/L produced an, I.E of 96% for mild steel. The extracts of Cactus cladode (Oulabbas et al., 2022) and Hibiscus sabdariffa (Ameer and Fekry, 2015) also showed promising IEs of 97.7% and 93.7% for mild steel in HCl media respectively. Many of these plant extracts adhere to the Langmuir adsorption isotherm, with either physical, chemical, or physiochemical modes of adsorption.

Moreover, copolymers synthesized from a combination of polymers have also been analyzed and show excellent inhibitive properties in many corrosive environments because of their combined or synergistic effects. Polymer grafting as a mode of synthesis has been demonstrated to avail the resultant polymers with novel and desirable qualities, as well as attribute enhancement for both natural and synthetic polymers (Liu et al., 2004; Srivastava et al., 2010; Umoren and Ekanem, 2010; Roy et al., 2014a; Geethanjali et al., 2014; Babaladimath et al., 2018). Some of the formulations of copolymers studied for their corrosion inhibition potential include; polyacrylamide + 5 mM iodide (Umoren et al., 2010), polyacrylamide and zinc (Manimaran et al., 2012), a formulation of polyethylene oxide, polyacrylamide, and carboxymethyl cellulose (Sedahmed et al., 1982), polyacrylamide-phenyl phosphate and 50ppm Zn^2+^ (Rajendran et al., 1998), the copolymer of acrylamide and 4-vinyl pyridine (Gao et al., 2002), polyaniline graft of xanthan gum (Babaladimath et al., 2018), etc. Other studies conducted involving formulations of copolymers of natural extracts for corrosion inhibition include lignin terpolymer at 1000 mg/L (Ren et al., 2008) in 10% HCl which produced 97% IE, 500 ppm of pectin-*g*-polyacrylamide (Geethanjali et al., 2014) in 3.5% NaCl with, IE, of 87%, 100 ppm of polyacrylamide-g-okra mucilage (Banerjee et al., 2012) in 0.5M H_2_SO_4_ showed an, IE of 97%, 100 ppm of fenugreek-g-polyacrylamide (Srivastava et al., 2010) in 0.5M H_2_SO_4_ had an, I.E., of 96%, xanthan gum-g-PANI with 100–200 ppm (Babaladimath et al., 2018) showed, IE of 94%, etc The copolymers mentioned above have high, IE for the respective metal surfaces, and at much lower concentrations than most plant extracts.

The polysaccharide of okra is known to have a good hydrophilic nature and is composed of complex polysaccharides. It is also widely available, inexpensive, and has proven to be effective in a variety of applications extending to various fields such as pharmaceuticals, food manufacturing, cosmetics, gelling agents, gum substitutes, etc. (Camciuc et al., 1998; Ahiakpa et al., 2014; Zaharuddin et al., 2014; de Alvarenga Pinto Cotrim et al., 2016). The grafted okra polysaccharide with different combinations of synthetic polymers has also been associated with a boost in reactive sites, flocculation properties, drag reduction, shelf life, thermal stability, and corrosion inhibition (Mishra and Pal, 2007; Anastasakis et al., 2009; Raj et al., 2020). Therefore, this polymer graft opens up a new avenue for investigation and application as a mild steel (MS) corrosion inhibitor in an HCl environment. Hot solutions of HCl are commonly used in oil well acidizing to improve well producibility and pickling. These, and other industrial practices, are known to accelerate corrosion in most industries, necessitating efficient remediation and inhibition (Lagrenee et al., 2001; Palmer et al., 2004; Rajeev et al., 2012; Bahadori, 2014; Muthukrishnan et al., 2014). In this paper, the corrosion inhibition potential of the polyacrylamide-okra graft was probed using the gravimetric method, and Box-Behnken design (BBD) from response surface methodology (RSM). The corrosion inhibition potential of the polyacrylamide-okra graft was compared against natural okra mucilage extract at 25 °C for 24 h. Thereafter, it was evaluated by designing and optimising the study using RSM while varying three independent variables: concentration, temperature, and immersion time. The study was optimized to obtain the lowest concentration, maximum immersion time, and temperature within the range of 25°C–65°C, to attain maximum, IE, FTIR, TGA, and FESEM analyses were also performed to supplement the corrosion data.

## 2 Experimental

### 2.1 Experimental design

There are a variety of factors that influence the corrosion inhibition potential of natural inhibitors. Some of the factors are the inhibitor concentration, surrounding temperature, immersion time, flow velocity, oxygen ingress, inhibitor solubility, etc. The relationship between three of these independent variables, their interactions, and their influence on inhibition efficiency (IE) was investigated in a limited time and range of experiments (Mehmood et al., 2018). This study employed a three-factor Box-Behnken design (BBD) to investigate and optimise the effect of variables; concentration (A), temperature (B), and time (C) on the inhibition efficiency (Y) of polyacrylamide-okra graft. The scope of variables A, B, and C were decided based on literature and screening experiments as; 100–500 ppm, 25°C–65°C, and 4–24 h respectively. The experimental data were collected through 15 runs, five center points, and ten factorial points, which were randomly performed and made to fit a second-order polynomial model to uncover the likely interactions in the design parameters using a response function as shown in Eq. 1:

$$\begin{array}{c} Y=\beta_{0}+\beta_{1}Y_{1}+\beta_{2}Y_{2}+\beta_{3}Y_{3}+\beta_{11}Y_{1}^{2}+\beta_{22}Y_{2}^{2}+\beta_{33}Y_{3}^{2}+\beta_{12}Y_{1}Y_{2}+\beta_{13}Y_{1}Y_{3}+\beta_{23}Y_{2}Y_{3} \end{array}$$ (1)

where Y represents the response value, β_J_ β_JJ_ β_JK_ which indicate the values of linear, quadratic, and interactive regression coefficients, respectively, and β_0_ is a constant.

All of the experimental data was analysed and made to fit a model using regression with Stat ease (Design Expert™ 13.0). Analysis of variance (ANOVA) was used to investigate the fitness and adequacy of the regression model. After that, the desirability function approach was deployed to optimize the copolymer’s corrosion inhibition ability. The model’s validity was then tested using a confirmation gravimetric test. The procedure was carried out three times to determine the relative error.

### 2.2 Materials and methods

**FIGURE 1:**
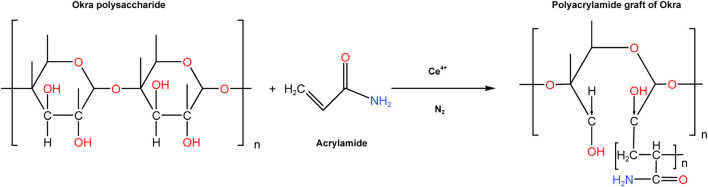
Mechanism for the synthesis of polyacrylamide-okra graft (Banerjee et al., 2012; Geethanjali et al., 2014).

Mature pods of okra were acquired from a local supermarket in Seri Iskandar. Acetone and 37% HCl were purchased from Merck and Fisher respectively. Prior to the extraction, the okra fruits were washed to remove any dirt from their surfaces. The okra pods were split to dispose of the seeds, after which they were blended with distilled water in a kitchen blender to create a slimy mixture that was then left overnight. The okra mucilage was then extracted using (de Alvarenga Pinto Cotrim et al., 2016) previously described precipitation method using acetone. The mucilage was then filtered, and dried for 24 h at 30°C before being crushed to powder with a porcelain grinder. A hybrid polymer was created from natural okra mucilage using free radical-initiated grafting. This is initiated from the polymer chain’s -OH groups using ceric ammonium nitrate and nitric acid as redox initiators. This method can be used to modify the structural motifs of the okra polysaccharide (Raj et al., 2020). This procedure was carried out with slight modifications to the work of many polymer scientists (Mino and Kaizerman, 1958; Deshmukh and Singh, 1986; Rath and Singh, 1997; Mishra et al., 2008). In an Erlenmeyer flask, 1 G of okra mucilage was dissolved in 200 mL of distilled water. The okra solution was transferred to a three-necked round-bottomed flask, sealed, and flashed with nitrogen (N_2_) gas for 20 min. Then acrylamide (2 g) was dissolved in distilled water (96.54 mL) and added to the okra mixture while stirring constantly. Following that, the solution was then bubbled with N_2_ for 30 min while being stirred. 0.1M ceric ammonium nitrate (CAN) solution was then prepared, and 3.46 mL of CAN was then injected into the flask using a hypodermic syringe to make a total reaction mixture of 300 mL. The N_2_ flashing was extended for 20 more minutes. By immersing the flask in silicon oil on a stirring plate, the reaction temperature was kept at 50°C. After another 2°h, the reaction was stopped by adding 0.5 mL of a solution of hydroquinone solution. With excess isopropanol, the product was precipitated and filter the reaction product. The homopolymer was then separated using a 1:1 mixture of N, N-dimethyl formamide, and acetic acid. The precipitate was slurried in acetone once more before being oven-dried at 30°C for 48 h. Figure 1 depicts the radical polymerization reaction that leads to the formation of polyacrylamide-okra graft. Mild steel coupons of composition Al, Ar, C, Cr, Fe, MN, Ni, P, S, Ag, S, and Zn of type S275 and dimensions 20 mm × 20 mm x 1.0 mm, were prepared for gravimetric tests by abrading with emery papers: 240, 320, 400, 600, and 800. They were degreased with ethanol to remove any grinding residues, rinsed in acetone, washed, and dried before weighing with an electronic balance.

### 2.3 Gravimetric test

Weight loss analysis was done in accordance with ASTM G31-72 using 100 & 200 mL of 1M HCl solution in the corrosive environment and at ambient temperature to evaluate the inhibitive properties of okra mucilage and polyacrylamide-okra graft respectively (Saxena et al., 2018). The concentration of okra mucilage extract varied from 200 to 25000 ppm, while that of polyacrylamide graft of okra varied between 20 and 800 ppm. The MS steel samples were then submerged in 1M HCl for 24 h, after which the samples were reweighed to calculate the weight lost. The tests were performed in triplicate, and the average results were obtained. The corrosion rates (CR) and inhibition efficiencies (IE) were calculated as illustrated in Eqs 2, 3 below (Loto et al., 2016; Oguntade et al., 2019; Edoziuno et al., 2020).

$$\begin{array}{c} Corrosion\;rate,CR\;\left(mmpy\right)=\frac{87.6*∆W}{DAT} \end{array}$$ (2)

$$\%\;IE=\frac{\left({∆W}_{1}-{∆W}_{2}\right)}{∆W_{1}}\times 100$$ (3)

where mmpy is millimeter per year, ∆W is weight loss (difference between coupons’ initial and final) (g), D is density (g/cm^3^), A is specimen area (cm^2^), T is time in hours, and ∆W_1_ and ∆W_2_ are the weight losses of mild steel in the absence and presence of the inhibitor.

### 2.4 Electrochemical tests

Electrochemical impedance spectroscopy (EIS) and potentiodynamic polarization (PDP) were carried out using a three-electrode cell system and an Autolab potentiostat. The cell system consisted of 200 mL of 1M HCl as the electrolyte, Ag/AgCl as the reference electrode, platinum as the counter electrode, and the working electrode (WE) as mild steel with dimensions (2 × 1 × 0.1 cm^3^) with exposed area of 1 cm^2^. All tests were carried out in unstirred conditions, without deaeration, and room temperature. Before electrochemical measurement, the WE was left immersed in the electrolyte for 30 min and the steady-state open circuit potential (OCP) was recorded. EIS measurements were carried out at a frequency range of 10 mHz–100 kHz, and amplitude of +5 mV. The polarization studies were carried out over a potential of +250 to −250 mV with respect to the OCP, and scan rate of 5mVs^-1^ (Banerjee et al., 2012; Bahlakeh et al., 2019). The corrosion current density (I_corr_) was determined from the intercept of the extrapolated cathodic and anodic Tafel slopes (β_c_ & β_a_) at the corrosion potential (E_corr_) (Srivastava et al., 2010). Fresh solutions and mild steel samples were used after each sweep. The impedance and polarization data were analysed using Nova 2.1.5 software (MetroOhm Autolab).

### 2.5 Characterization of the polymers

The modified compound’s functional groups were identified using Fourier transform infrared spectroscopy (FTIR) at wavenumbers ranging from 4,000 to 500cm^-1^. The FTIR spectra were obtained using the attenuated total reflection (ATR) method using a PerkinElmer Spectrum 400 spectrometer. The thermal characteristics of the extract were analyzed by Thermal gravimetric analysis (TGA) methods. TGA was conducted using the STA600 PerkinElmer, in the range of 30°C–900°C by increments of 10°C/min in an ambient environment.

### 2.6 Morphological study

Field Emission Scanning Electron Microscopy (FESEM) at 5,000X resolution was used to examine the surface characteristics of the original, corroded, and inhibited MS metal surfaces. The MS samples were submerged in HCl solution for 24 h with and without 800 ppm of polyacrylamide-okra graft. The images of the various forms of MS surfaces were then taken along with the elemental composition using Energy Dispersive X-Ray Analysis (EDX).

## 3 Results and discussion

### 3.1 Gravimetric studies

**FIGURE 2:**
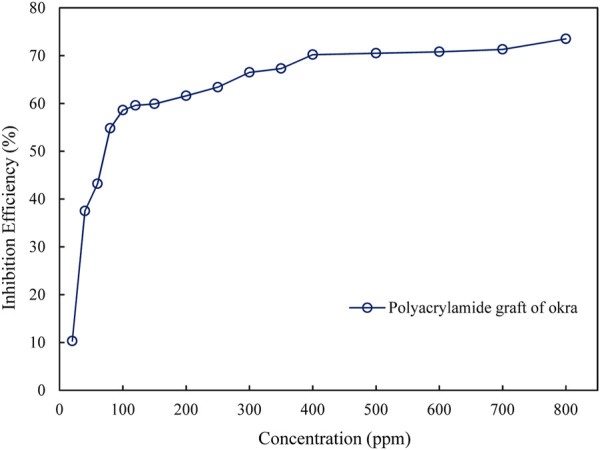
Inhibition performance of polyacrylamide graft of okra.

**FIGURE 3:**
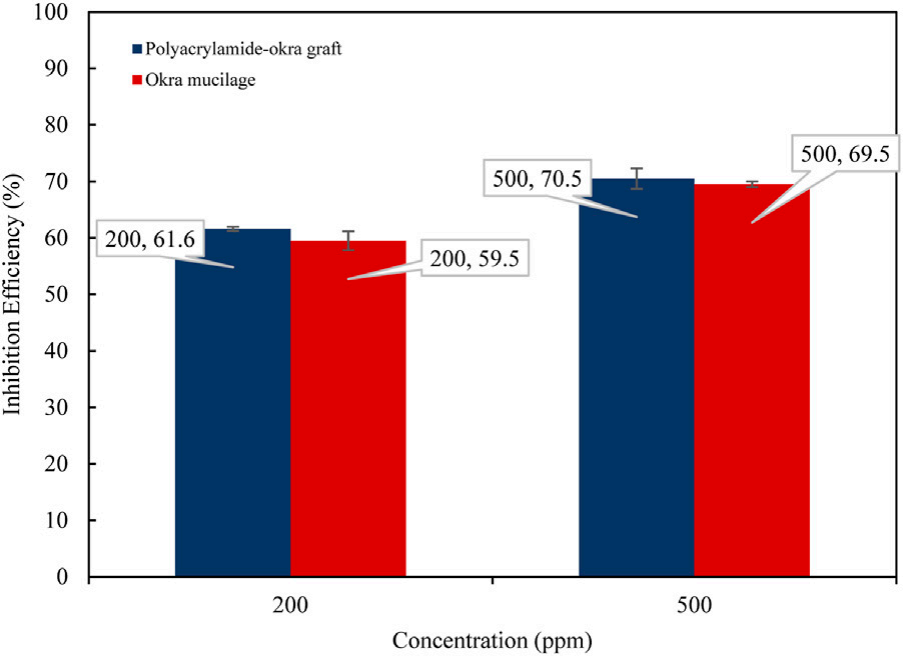
Comparison of, I.E., performance of okra mucilage powder and polyacrylamide-okra graft at 25 °C for 24 h.

The gravimetric corrosion data for okra mucilage and polyacrylamide-okra graft, at ambient temperatures for 24 h are presented in Table 1. The results show that increasing concentrations of okra mucilage and polyacrylamide-okra graft increased inhibition efficiency from 59.5% to 89% and 54.8%–73.5% respectively as shown in Figure 2. This effect is predicted to be due to the increase in the quantity of the adsorbed inhibitor molecules on the metal surface (Chigondo and Chigondo, 2016; Heidarshenas et al., 2020). The rate of increase in, I.E., was slower at higher inhibitor concentrations for both extracts. This is because as the inhibitor concentration increases, the polymer molecules cover most of the active corrosion sites such that further addition of the inhibitor has minimal effect on the corrosion rate or inhibition efficiency (Odewunmi et al., 2020). Further increases in inhibitor concentration for both extracts are not included as they had a slight effect on the, IE due to decreasing inhibitor solubility at very high concentrations. Non-etheless, the high inhibition performance of okra mucilage is more correlated to higher concentrations than for polyacrylamide-okra graft. At inhibitor concentrations of 200 and 500 ppm, the polyacrylamide-okra graft displayed a superior performance (61.6% & 70.5% IE) compared to okra mucilage extract (59.5% & 69.5% IE) as shown in Figure 3. Moreover, the copolymer’s better performance can be attributed to grafting with polyacrylamide that enhances its stability and adsorption capabilities, even though the polymer had a limited solubility in HCl at very high concentrations.

**TABLE 1:** Weight loss analysis for mild steel with different concentrations of okra mucilage and polyacrylamide-okra graft extracts at 25 °C for 24 h.

Okra mucilage			Polyacrylamide-okra graft		
Concentration (ppm)	Corrosion rate (mmpy)	IE (%)	Concentration (ppm)	Corrosion rate (mmpy)	I.E (%)
Blank	0.0053	—	Blank	0.00528	—
200	0.0021	59.5	20	0.0047	10.3
500	0.0016	69.5	40	0.0033	37.5
1,000	0.0015	75.7	60	0.003	43.2
1,500	0.0012	78.3	80	0.0024	54.8
2,000	0.0006	87.9	100	0.0022	58.6
2,500	0.0006	89.0	150	0.0022	59.9
5,000	0.0060	91.7	200	0.0020	61.6
10,000	0.0037	94.8	250	0.0019	63.4
15,000	0.00370	94.9	300	0.0018	66.5
20,000	0.0030	95.9	350	0.0017	67.3
25,000	0.0029	96.0	400	0.0016	70.2
			500	0.0016	70.5
			600	0.0015	70.8
			700	0.0015	71.3
			800	0.0014	73.5

The presence of more bonding locations in the graft polymer, N_2_ and O_2_ atoms with lone pairs in the structure, along with the -NH_2_ functional group from the acrylamide polymer also equip the new compound with better adsorption abilities towards the MS vacant d-orbitals (Srivastava et al., 2010; Banerjee et al., 2012). To further analyse the influence of factors such as concentration, temperature, and immersion time on the, I.E., of the polyacrylamide-okra graft, the study was designed with lower concentration (100–500 ppm), temperature between 25°C–65°C and immersion time from 4—24 h using RMS’s Box-Behnken design.

### 3.2 Adsorption isotherms

Adsorption isotherms provide useful information about how corrosion inhibitors interact with the metal surfaces they are designed to protect (Ituen et al., 2017). The parameters used to determine this are surface coverage (θ) and concentration of the inhibitor (C_inh_). This corrosion inhibition data was then used to investigate several adsorption isotherms. For this study, the Temkin and Langmuir adsorption isotherms were developed, using their relationships between surface coverage and inhibitor concentration as shown in Eqs 4, 5 respectively, to show the inhibitor-adsorption behaviour.

$$\begin{array}{c} \theta =\frac{1}{f}{lnK}_{ads}.C_{inh} \end{array}$$ (4)

$$\begin{array}{c} \frac{C_{inh}}{\theta }=\frac{1}{K_{ads}}+C_{inh} \end{array}$$ (5)

where *C*_*inh*_ is the inhibitor concentration, θ is the surface coverage, 1/f is the slope of the graph, and *K*_*ads*_ is the equilibrium constant of the adsorption process.

**FIGURE 4:**
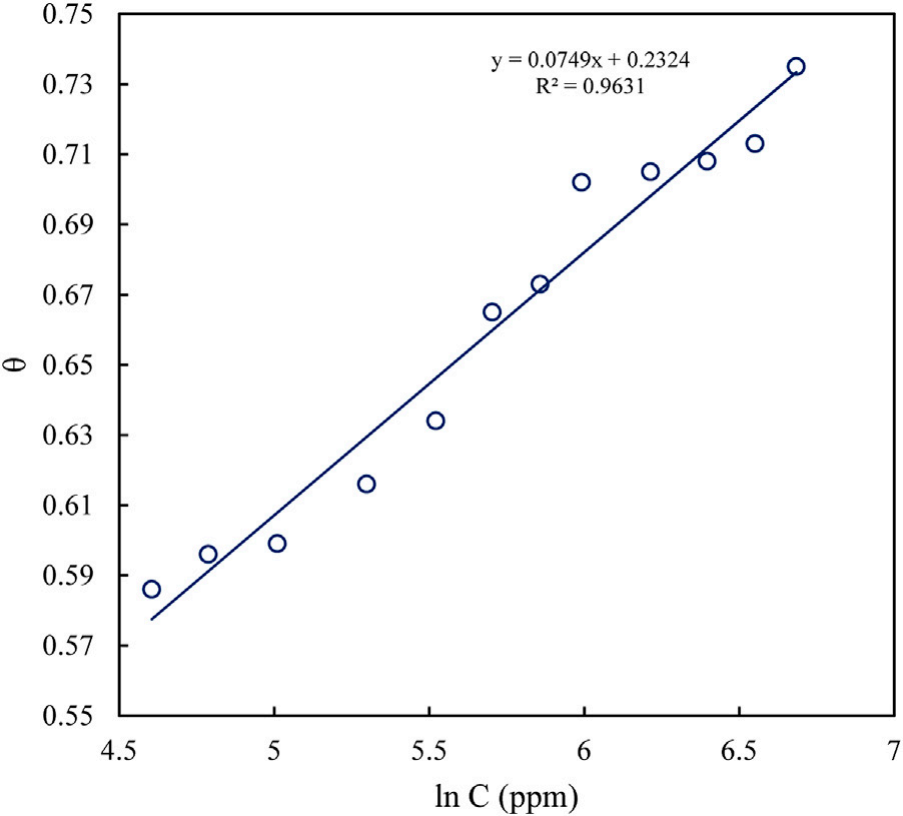
Temkin adsorption isotherm model fitted for polyacrylamide-okra graft at 25 °C.

**FIGURE 5:**
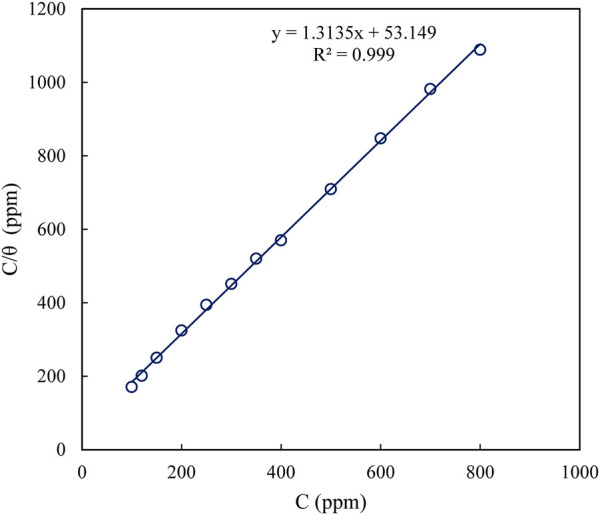
Langmuir adsorption isotherm model for polyacrylamide-okra graft at 25 °C.

According to the correlation coefficients of the plots, it was observed that the adsorption behavior of polyacrylamide-okra graft in 1M HCl solution on the MS surface was best explained by the Langmuir adsorption isotherm. This is shown by the linear relationship observed by plotting C_inh_/θ against C_inh_ (Figure 4) with a correlation coefficient (R^2^) of 0.999, in comparison to the Temkin (R^2^ = 0.9631) (Figure 5). The K_ads_ value was determined as 1.88 × 10^−2^ ppm^-1^ from the intercept of the Langmuir adsorption graph. This high value suggests stronger interaction between the inhibitor molecules and mild steel (Zarrok et al., 2012). The line’s slope is near unity (1.3135) which validates the adherence of the adsorption behaviour to the Langmuir isotherm (Arthur and Abechi, 2019; Al-Baghdadi et al., 2021). R^2^ was used to determine the experimental data’s fit to the adsorption isotherm. Correspondingly, the Gibbs free energy of activation (∆G^°^_ads_) was determined from the equilibrium constant (K_ads_) as −24.4 kJ/mol using Eq. 6:

$$\begin{array}{c} ∆G_{ads}^{{}^{\circ}}=-RT\ln\left(1\times 10^{6}K_{ads}\right) \end{array}$$ (6)

where R is the molar gas constant (kJ.K^−1^mol^-1^), T is the absolute temperature (K), K_ads_ is the adsorption equilibrium constant (ppm^-1^), and 1×10^6^ is the molar concentration of water in the solution in ppm.

The negative value of ∆G^°^_ads_ is indicative of the spontaneity and stability of the adsorption phenomena (El-Haddad, 2013; Roy et al., 2014b). Values of ∆G^°^_ads_ > −20 are representative of physical adsorption, which occurs because of electrostatic interactions. ∆G^°^_ads_ < −40 is representative of chemical adsorption, which is a reaction between unshared electron pairs of the inhibitor molecules and unoccupied d-orbitals, while values between −40 and −20 represent a mixture of the two modes of adsorption, commonly referred to as physio-chemisorption adsorption (Saxena et al., 2018; Al-Baghdadi et al., 2021). The ∆G°_ads_ result obtained is therefore characteristic of physio-chemical adsorption of the polyacrylamide-okra graft on the MS surface.

### 3.3 Electrochemical analysis

#### 3.3.1 Electrochemical impedance spectroscopy

**FIGURE 6:**
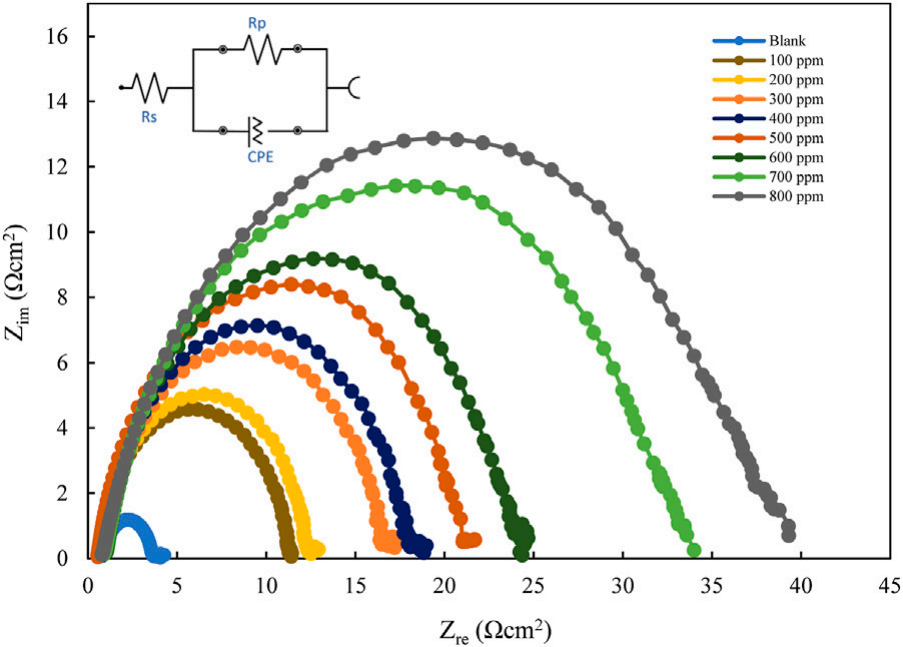
Nyquist plots for mild steel in 1 M HCl with varying concentrations of polyacrylamide graft of okra.

**FIGURE 7:**
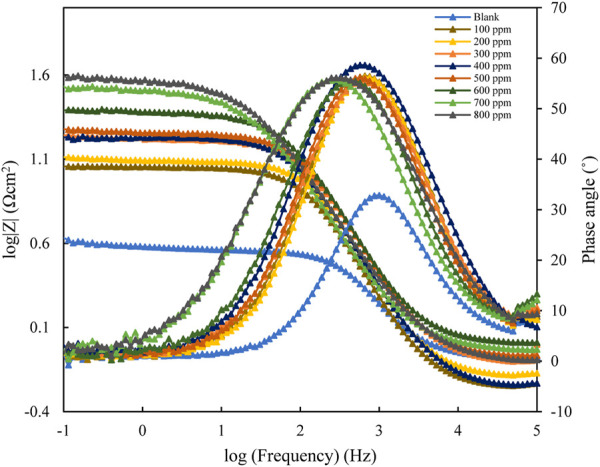
Bode and phase angle plots for mild steel in 1 M HCl with varying concentrations of polyacrylamide graft of okra.

The EIS results for mild steel in 1M HCl, using polyacrylamide graft of okra inhibitor obtained, at 25°C are presented in Figure 6; Figure 7 below. The EIS results obtained could be interpreted in terms of a simple electrical circuit model, which is a combination of R_s_ (solution resistance), CPE (constant phase element), and R_ct_ (charge transfer resistance). In Figure 6, typical Nyquist plots with single semicircles are observed varying with concentration along the real impedance. The single semi-circles, which represent a single charge transfer process correspond to one capacitive loop (Idris et al., 2013). These impedance values increase with inhibitor concentration, without changing the impedance profile at all concentrations. This is evidence of a similar adsorption mechanism. Each plot is also depressed, which shows surface heterogeneity due to the microscopic roughness of the metal surface and the adsorption of the inhibitor on it (Srivastava et al., 2010; Roy et al., 2014a). The respective profiles are confirmed by the bode plot analysis (Figure 7), whose single-phase horn shows the dominance of charge transfer in the corrosion process of mild steel (Zhang et al., 2020).

The R_ct_ values are calculated from the difference in impedance at lower and higher frequencies. In Table 2, an increase in inhibitor concentration is observed to bring about a corresponding increase in R_ct_ at the metal-electrolyte interface. The R_ct_ values were used to compute the Inhibition efficiency using the relationship below (Eq. 7):

$$\begin{array}{c} IE=\frac{R_{ct}-R_{ct,0}}{R_{ct}}\times 100 \end{array}$$ (7)

where R_ct,0,_ and R_ct_ are the charge transfer resistance values in the absence and the presence of an inhibitor respectively. The impedance values of CPE can be calculated by the Eq. 8:

$$\begin{array}{c} Z_{CPE}=\frac{1}{Y_{o}{\left(j\omega \right)}^{n}} \end{array}$$ (8)

where *Y*_*o*_ is the CPE constant, *j* is the imaginary number, ω is the angular frequency (*ω = 2πf, f* is the frequency), and *n* is the CPE exponent. The constant *n* which serves to measure the surface heterogeneity is usually between 0.9 and 1. If *n* is equal to 1, *Z*_*CPE*_ tends an ideal capacitor (Instruments, 2007), with double-layer capacitance (*C*_*dl*_). The *C*_*dl*_ value can be estimated from Eq. 9:

$$\begin{array}{c} C_{dl}={\left(Y_{o}{R_{ct}}^{1-n}\right)}^{\frac{1}{n}} \end{array}$$ (9)

**TABLE 2:** Electrochemical Impedance spectroscopy corrosion parameters for mild steel in 1M HCl with polyacrylamide graft of okra at 25°C.

Conc (ppm)	R_s_ (Ωcm^2^)	R_ct_ (Ωcm^2^)	Y_o_ (μΩcm^2^s^-n^)	n	C_dl_ (μFcm^-2^)	I.E., (%)
Blank	0.85	2.7643	127.98	0.903	54.89	—
100	0.57	10.802	113.16	0.89	50.84	74.4
200	0.68	11.689	96.047	0.91	47.2	76.4
300	0.79	15.889	86.955	0.87	32.50	82.6
400	0.90	16.952	85.568	0.89	38.41	83.7
500	0.50	20.523	133.82	0.86	51.24	86.5
600	0.97	23.066	96.02	0.85	31.86	88.0
700	0.69	32.689	178.54	0.78	41.71	91.5
800	0.55	37.543	158.67	0.77	33.0	92.6

Table 2 shows the values of R_s,_ *R*_*ct*_ Y_o_ n, and C_dl_ from the impedance data. R_ct_ values increase gradually with a concurrent general decrease in *C*_*dl*_ values which is evidence of the formation of a protective film and a decrease in the available active sites for the corrosion reaction. This shows that the inhibitor molecules replaced the water molecules in the vicinity of the metal surface (Azzam et al., 2018; Mobtaker et al., 2021). Furthermore, a decrease in capacitance suggests that there is a drop in the local dielectric constant, and an increase in the thickness of the electrical double layer (Zarrok et al., 2012). However, the *C*_*dl*_ values do not follow a regular trend, which may be an effect of the limited solubility of the natural polymer in the acidic medium at such high concentrations (Banerjee et al., 2012). This is because capacitance is inversely proportional to *C*_*dl*_ thickness (Helmholtz equation (Eq. 10) (Idris et al., 2013).

$$\begin{array}{c} C_{dl}=\frac{\varepsilon \varepsilon_{o}A}{d} \end{array}$$ (10)

where *d* is the thickness of the latter, *A* is the surface area of the electrode, ε is the dielectric constant of the medium, and *e*_o_ is the vacuum permittivity.

The increase in, IE with the concentration of the okra mucilage powder inhibitor is because of the availability of more molecules for adsorption at higher concentrations. It is therefore concluded from the impedance measurements that the addition of polyacrylamide graft of okra into the 1M HCl corrosive solution caused an increase in charge-transfer resistance, and, IE, and a general decrease in the double-layer capacitance (C_dl_). These data were in good agreement with the corrosion weight loss data.

#### 3.3.2 Potentiodynamic polarisation

**FIGURE 8:**
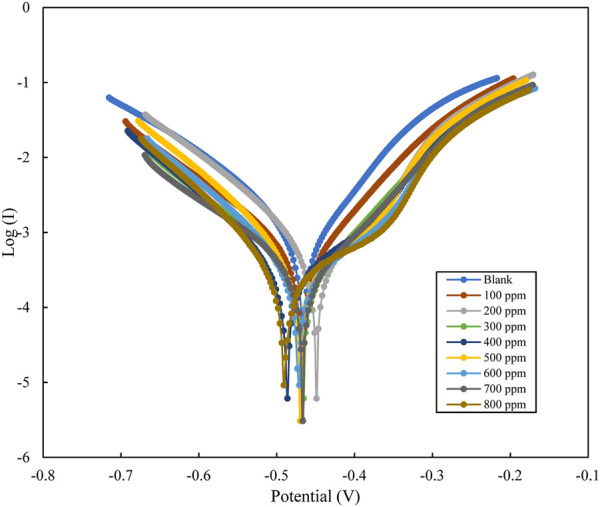
Potentiodynamic polarization plots for mild steel in 1M HCl with varying concentrations of polyacrylamide graft of okra inhibitor.

Polarization analysis for mild steel in 1M HCl using polyacrylamide graft of okra, with inhibitor concentration varying from 100–800 ppm. The tafel plots are presented in Figure 8, which appear as parallel lines, therefore evidence of activation hydrogen evolution (Bentiss et al., 1999). The other tafel parameters: (*E*_*corr*_ *, I*_*corr*_ *, β*_*a*_ *, β*_*c*_) obtained by extrapolation of the tafel curves are detailed in Table 3. The experimental tafel data shows a decrease in the *I*_*corr*_, from 531 to 117 μAcm^-2^ with respect to the blank, as the concentration of the inhibitor increased to 800 ppm. Similarly, values of *E*_*corr*_ were observed to change with respect to the blank with changing inhibitor conditions. The observed shift with respect to concentration is in both directions, and the *E*_*corr*_ difference is <85 mV. This is a sign of effects on both the cathodic and anodic reactions, with the cathodic influence being more. This is characteristic of mixed-type inhibitors (Srivastava and Singh, 2010; Roy et al., 2014a). The small shifts in small shifts in the β_a_, and β_c_ tafel slopes values are evidence that the inhibition mechanism occurs by the blocking of the reactive sites, and not alteration of the corrosion mechanism (Azzam et al., 2018; Zhang et al., 2020; Mobtaker et al., 2021). The inhibition efficiency was calculated from the measurements of I_corr_ using the relationship in Eq. 11 below:

$$\begin{array}{c} IE=\frac{I_{corr,0}-I_{corr}}{I_{corr,0}}\times 100 \end{array}$$ (11)

where I_corr,0,_ and I_corr_ are the corrosion current densities in the absence and the presence of polyacrylamide graft of okra inhibitor respectively.

**TABLE 3:** Potentiodynamic polarisation measurements for mild steel in 1M HCl with varying concentrations of polyacrylamide graft of okra at 25°C.

Conc (ppm)	*-E*_*corr*_ (mV)	*I*_*corr*_ (μAcm^-2^)	*β*_*a*_ (mV/dec)	*β*_*c*_ (mV/dec)	CR (mmpy)	I.E., (%)
Blank	459.36	530.53	71.883	92.635	6.2444	—
100	459.62	375.94	84.477	116.91	4.4249	29.13
200	444.45	292.13	96.233	82.527	3.3707	44.93
300	456.37	218.57	87.024	118.57	2.5726	58.8
400	485.38	209.25	136.69	99.94	2.4629	60.56
500	473.46	189.41	123.96	61.19	2.2294	64.29
600	472.99	178.88	127.09	75.194	2.1054	66.28
700	469.6	158.67	92.472	72.735	1.8676	70.09
800	496.89	117.23	110.49	42.324	1.3798	77.9

The values of inhibition efficiency increased from 29% to 78% with an increase in inhibitor concentration from 100–800 ppm, and the corrosion rate decreased from 6.2 to 1.4 mmpy. This further explains the inhibitory action of the polyacrylamide graft of okra extract for mild steel in HCl, which is predicted to be due to the combination of multiple hydroxyl groups and oxygen atoms in the inhibitor, availability of lone pairs, and the strong amide groups from the synthetic polymer. This performance is in good agreement with the weight loss and impedance data recovered.

### 3.4 Statistical analysis

#### 3.4.1 Response surface methodology

**FIGURE 9:**
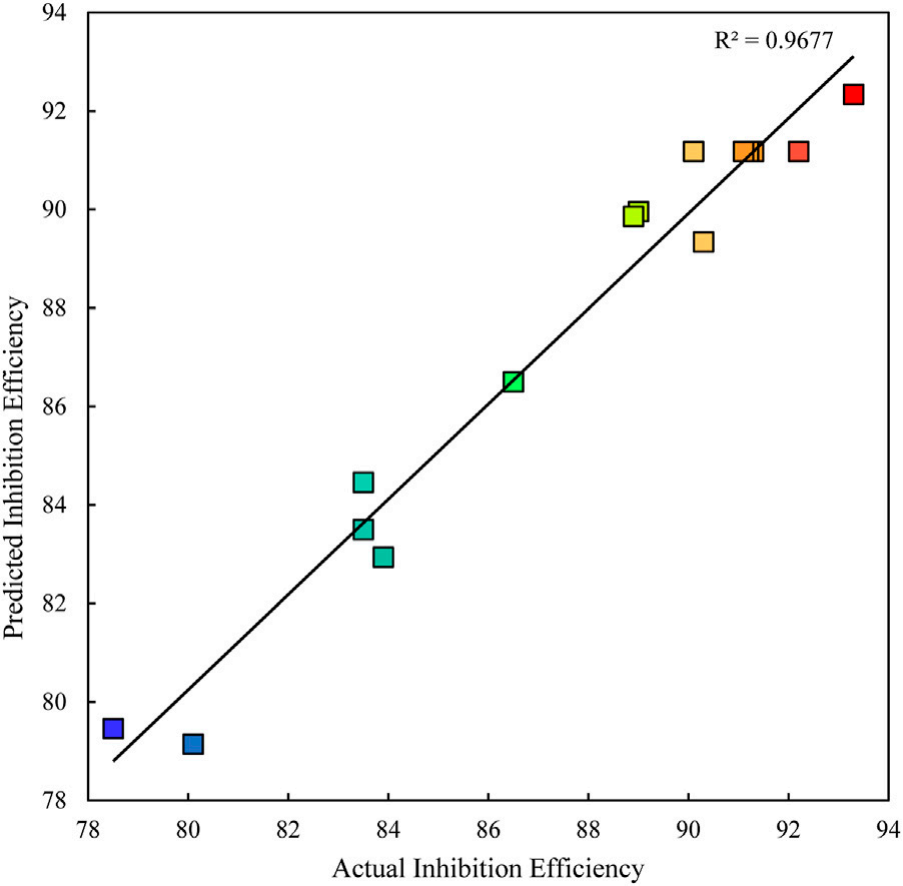
Predicted *versus* Actual inhibition efficiency.

To investigate the effect of various factors: concentration, temperature, and immersion time on the, IE by polyacrylamide-okra graft, a set of experiments was conducted based on an experimental design from the Box-Behnken as presented in Supplementary Table S1. A second-order quadratic regression model was used to fit the statistical data, and the summary of the model fitting is presented in Supplementary Table S2. Figure 9 shows the correlation between predicted and experimental values of inhibition efficiency. The regression equation for inhibition efficiency obtained from RMS in terms of actual factors is shown in Eq. 12.

$$\begin{array}{c} IE=49.092+0.044225A+0.918125B+1.08883C-0.000125AB\\ +0.00025AC-0.004625BC-5.35e^{-05}A^{2}-0.0066B^{2}-0.03065C^{2} \end{array}$$ (12)

where A is concentration, B is temperature and C is immersion time.

**FIGURE 10:**
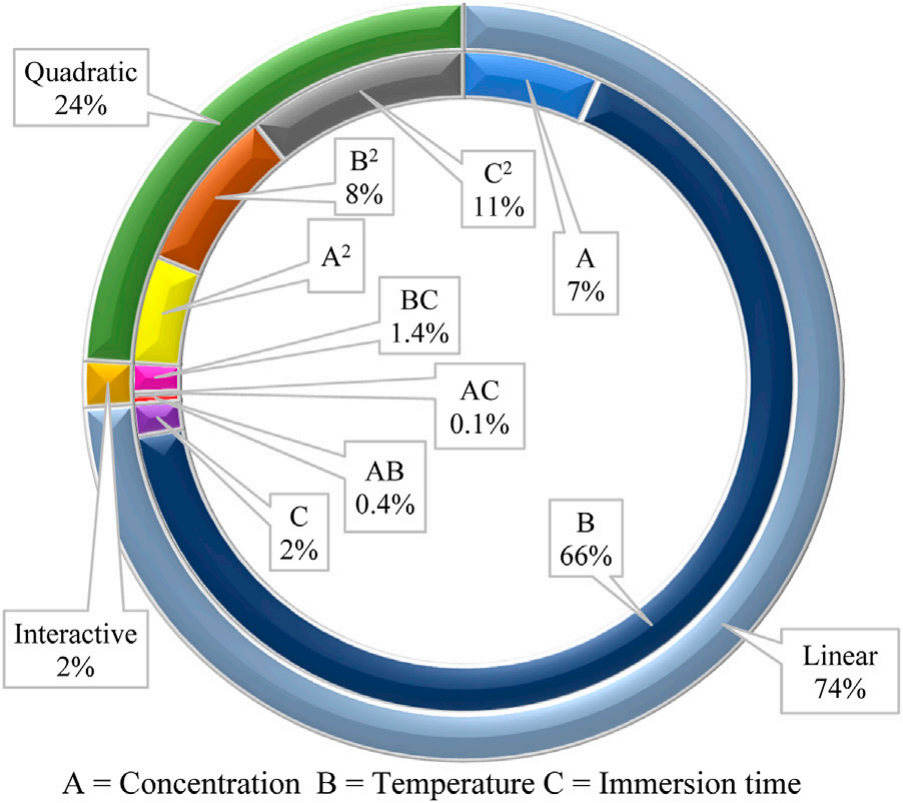
Influence of various factors in linear, interactive, and quadratic terms on corrosion inhibition efficiency of okra mucilage powder.

The quadratic model’s fitness and predictability are further evaluated by analyzing the variance. The statistical analysis and ANOVA for the quadratic model are shown in Table 4. The model has a significant F-value (16.58) and *p*-value (*p* = 0.0032), implying that it is significant, and so are the model terms. The large F-value suggests the presence of noise, and only occurs 0.32% of the time. The *p*-value of 0.0218 and F-value of 13.31 for the Lack of Fit indicate that the Lack of Fit is significant, which is undesirable. This Lack of Fit F-value has a 2.18% chance of occurring. Even so, the R^2^ and adjusted R^2^ values are calculated at 0.97 and 0.91, respectively, indicating that the sample factors and size are adequate to represent the model. The coefficient of variation (CV) is 1.59%, which because it is low, is a sign of a high level of dependability of the results (Maran et al., 2015). The signal-to-noise ratio is used to calculate adequate precision. Values not less than 4 are preferred to establish the model’s suitability (Balasubramani et al., 2015). In the current survey, the adequate precision is 11.64, indicating a sufficient signal from the model. From Table 4, *p*-values <0.05 are considered significant for the interpretation of model terms. In this case, linear terms (A, B), and quadratic terms (B^2^, C^2^) are significant model terms. Significant model terms have a high influence on the inhibition efficiency of the polyacrylamide-okra graft. The effect of parameters on inhibition efficiency in terms of linear, interactive, and quadratic model terms is depicted in Figure 10 in relation to ANOVA (sum of squares of all factors). The linear terms show a paramount influence of 74% on inhibition efficiency, then the quadratic terms, with a modest influence of 24%, and finally the interactive terms with the lowest impact (2%) on inhibition efficiency. As a result, it appears that temperature (B = 65.7%) has the greatest influence on corrosion inhibition by polyacrylamide-okra graft, followed by inhibitor concentration (A = 6.7%). The immersion time has the least influence (C = 1.6%) as confirmed by a *p*-value of 0.2192. Nevertheless, its quadratic term has a significant *p*-value (*p* = 0.0145) and a distinct influence (C^2^ = 10.8%). The quadratic term for temperature also has a significant influence (B^2^ = 8%). For the interactive terms, none of the interactions seemed to be significant to the corrosion inhibition process [*p*-values >0.05].

**TABLE 4:** ANOVA and fit statistics for the quadratic regression model.

Source	Sum of squares	Df	Mean square	F-value	*p*-value	
Model	287.66	9	31.96	16.58	0.0032	significant
A-Concentration	16.00	1	16.00	8.30	0.0346	
B-Temperature	157.53	1	157.53	81.71	0.0003	
C-Time	3.80	1	3.80	1.97	0.2192	
AB	1.0000	1	1.0000	0.5187	0.5036	
AC	0.3333	1	0.3333	0.1729	0.6948	
BC	3.42	1	3.42	1.78	0.2402	
A^2^	12.63	1	12.63	6.55	0.0507	
B^2^	19.23	1	19.23	9.97	0.0252	
C^2^	25.92	1	25.92	13.44	0.0145	
Residual	9.64	5	1.93			
Lack of Fit	7.41	1	7.41	13.31	0.0218	significant
Pure Error	2.23	4	0.5570			
Cor Total	297.30	14				
Std. Dev	1.39		R^2^	0.97		
Mean	87.56		Adjusted R^2^	0.91		
C.V. %	1.59		Adequate Precision	11.64		

#### 3.4.2 Effect of concentration, temperature and immersion time on inhibition efficiency

**FIGURE 11:**
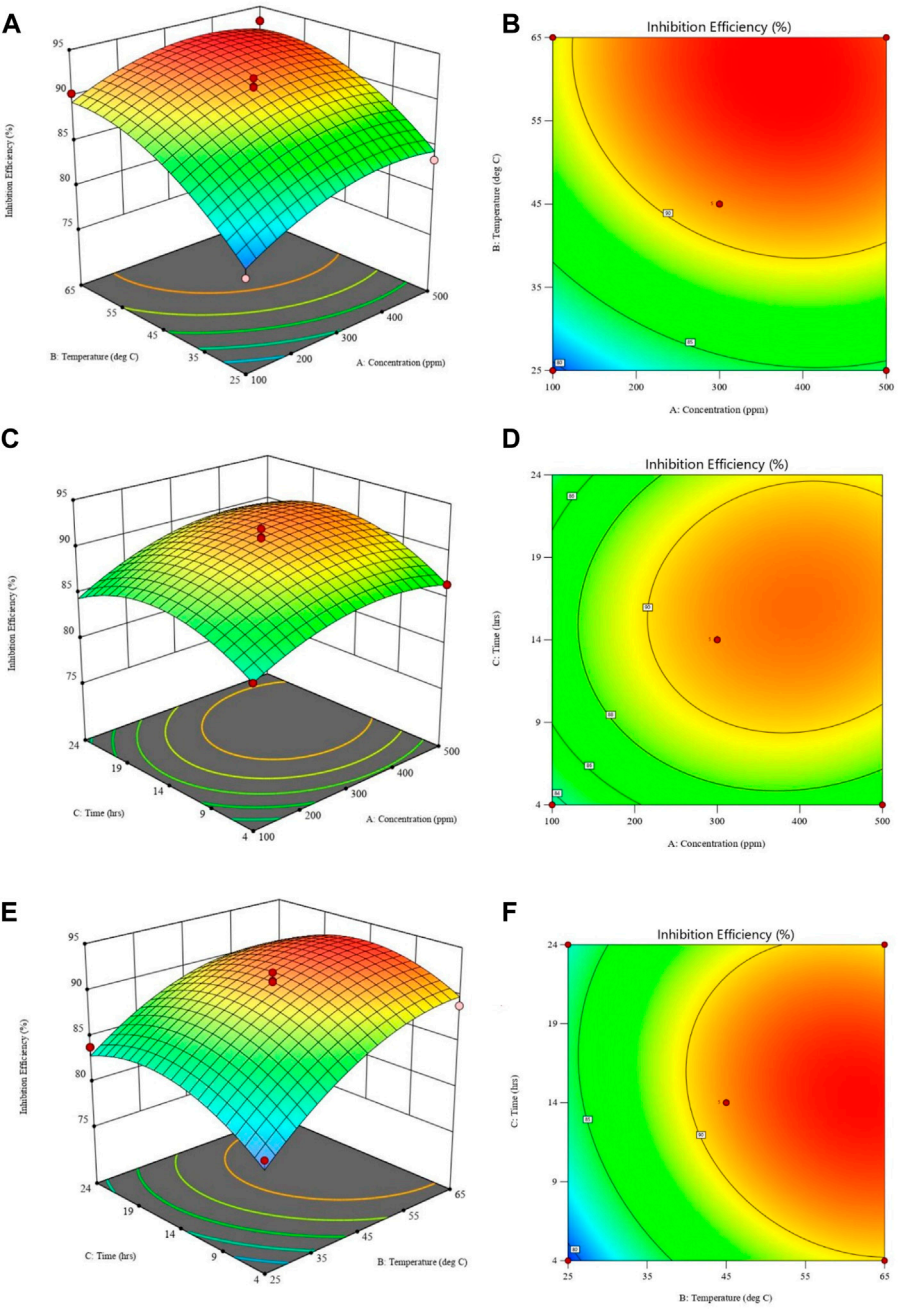
Response surface plots (left) and contour plots (right) for inhibition efficiency against: **(A,B)** temperature and concentration; **(C,D)** time and concentration; **(E,F)** time and temperature.

The impact of various factors on inhibition efficiency is investigated further using three-dimensional response surface and contour plots. Each plot is a combination of two factors while the third is kept constant as shown in Figures 11A–F. Figure 11A shows that, IE increases with temperature and inhibitor concentration, at that at each temperature there is an increase in, IE with inhibitor concentration. However, from Figure 11B, the interaction between temperature and concentration shows a significant effect on, IE, with the effect of temperature shown to be greater than that of concentration. The greater availability of inhibitor molecules on the metal surface to form a barrier and thus inhibit corrosion is responsible for the increase in, IE with inhibitor concentration. Some cases in literature (Nahlé et al., 2012; Khaleel et al., 2018), show that usually corrosion rates increase with temperature, even in the presence of inhibitors due to thermal degradation, desorption of the inhibitor, increased transport of reactants, and reduction in oxygen solubility that allows the cathodic reaction to occur. This was not the observed case with polyacrylamide-okra graft, whose action can be explained by the physio-chemisorption mode of the adsorption, as earlier mentioned in the adsorption analysis. That is, as the temperature rises, the inhibitor and MS metal’s ability to form coordinate bonds increases (Quy et al., 2019). This occurs through the donation of lone electron pairs of sulfur and nitrogen to empty orbitals of the iron atoms. Moreover, this improved performance can be attributed to the grafting of the natural polymer to polyacrylamide, which increased the number of lone pairs and enhanced the polymer’s stability and adsorption capabilities even at high temperatures. From the response surface plots in Figures 11C, D, it is shown that the immersion time has a minimal effect on the IE due to minimal changes in the IE across the time changes. However, as the inhibitor concentration increases, the effect of immersion time on IE increases slightly as depicted in Figure 11D. This is because, at higher concentrations, the rate of inhibitor adsorption is slightly increased due to an increase in the availability of inhibitor molecules. In Figures 11E, F, the dominant influence of temperature over immersion time is displayed as IE generally increases more with the increase in temperature than with an increase in immersion time.

**FIGURE 12:**
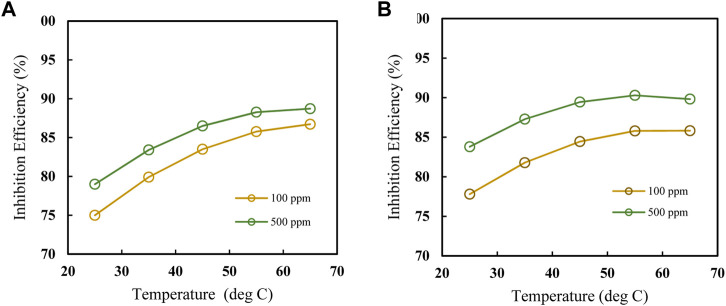
Effect of temperature on IE at 100 and 500 ppm at **(A)** 4 h and **(B)** 24 h.

**FIGURE 13:**
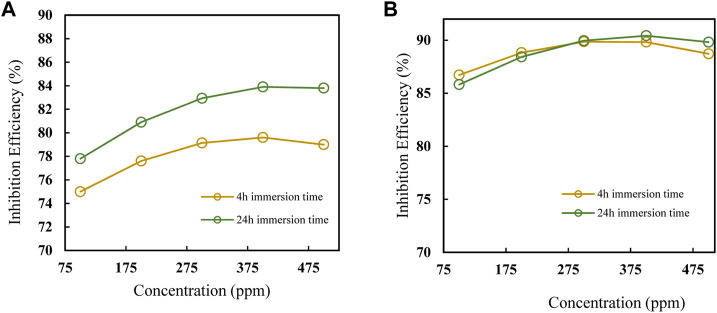
Effect of concentration on IE, at 4 h and 24 h for **(A)** 25°C and **(B)** 65°C.

**FIGURE 14:**
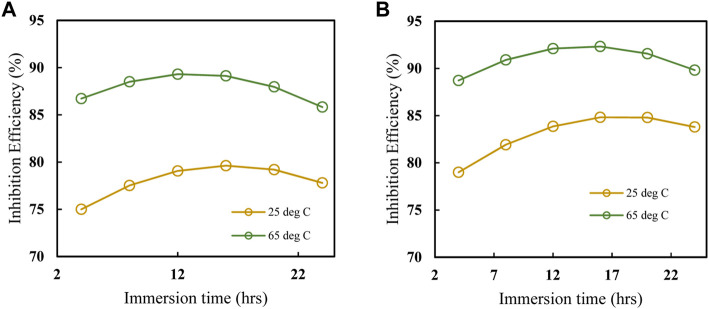
Effect of immersion time on IE, at 25°C & 65°C for **(A)** 100 and **(B)** 500 ppm.

Plots showing the influence of temperature, concentration, and immersion time on the inhibition efficiency of the polyacrylamide-okra graft were also developed using the regression equation (Eq. (12)) obtained from RMS in terms of actual factors. From Figures 12A, B, at both 100 and 500 ppm inhibitor concentrations, there is a noticeable increase in, I.E., with the temperature at the 4- and 24- immersion times as earlier depicted in Figure 12A. The effect of immersion time between the two plots is very minimal, however, there is a significant better IE performance of the 500ppm inhibitor at the 24 h immersion time compared to 4 h. The effect of concentration on, I.E., (Figures 13A, B) is observed to be positive, at the 4h and 24 h immersion times, and in the 25 & 65 °C plots. In addition, the inhibition efficiencies differ slightly in both plots due to the effect of different immersion times. This is further evidence that immersion time has a negligible effect on the inhibitor’s performance. Furthermore, the inhibitor proved to have the highest IE performance at 65°C, with the effect of immersion time almost negligible than at 25°C. This follows the same trend as in Figures 11A, C. Likewise, Figures 14A, B depict the effect of immersion time on inhibition efficiency at 25°C and 65°C for 100 and 500 ppm inhibitor concentrations. There is a slight variation in IE with immersion time observed for both concentrations, however, the IEs at 25°C are much lower than those at 65°C in both plots. This supports the fact that higher IEs are observed at higher temperatures in the presence of the inhibitor, as shown by the plots in Figures 11C, D above. To optimize the inhibition performance of the inhibitor, desirable parameters may be set using the numerical optimization tool in Stat ease (Design Expert™ 13.0). The desirable parameters were to lower the inhibitor concentration and increase the immersion time while maintaining the reaction temperature between 25°C and 65°C.

#### 3.4.3 Optimization process and validation of RSM model

**FIGURE 15:**
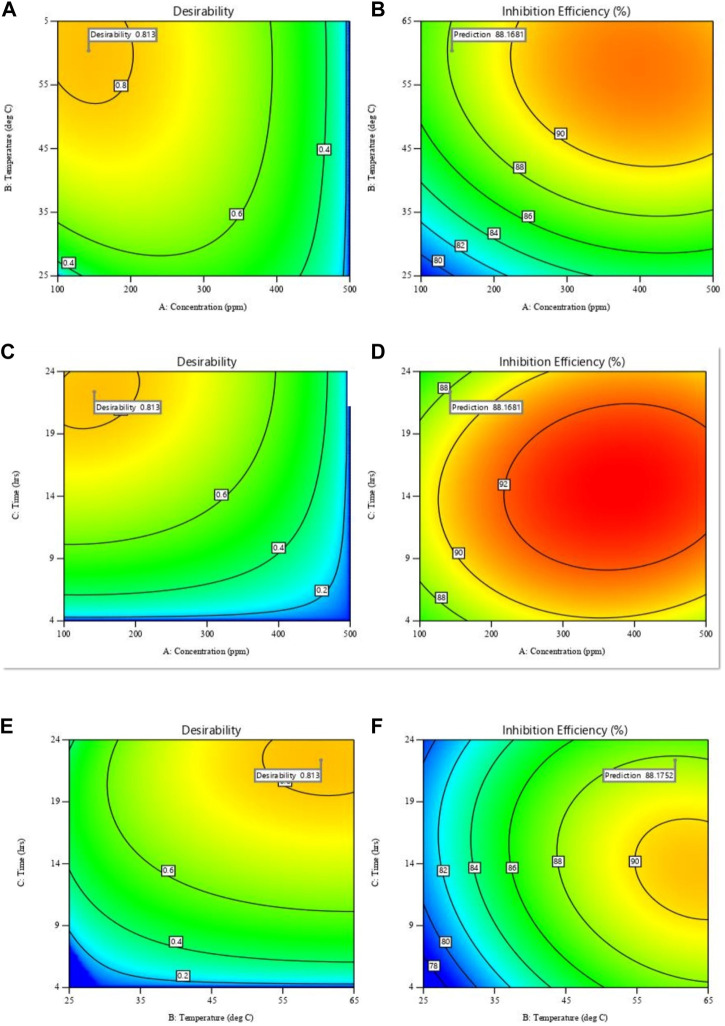
Contour plots for desirability (left) and inhibition efficiency (right) against: **(A,B)** temperature and concentration; **(C,D)** time and concentration; **(E,F)** time and temperature.

Using the desirability function approach, the developed model was used to optimise the design parameters to obtain the desired corrosion inhibition objectives. This was performed by transforming the estimated response into a dimensionless value known as desirability. The desirability function employed is dependent on the objectives of the optimization (Amdoun et al., 2018). The set objectives for this optimization were a minimum level of inhibitor concentration, maximum immersion time, and temperature in the range of 25°C–65°C to attain maximum inhibition efficiency. The optimization constraints are summarized in Table 5. One solution, with a desirability of 0.813 was obtained. The optimized corrosion inhibition solution offered an optimum level of inhibitor concentration at 142.3 ppm, temperature at 60.°C, and immersion time of 22.4 h to give a predicted, IE of 88.2%. The contour plots in Figure 15 show the relationship between desirability and inhibition efficiency with the various independent parameters. It is observed from Figure 15A that, based on the objective to minimize the inhibitor concentration, higher desirability is obtained at lower concentration values, although the inhibition efficiency increases with concentration (Figure 15B). Additionally, it is already established that temperature has a positive relationship with inhibition efficiency, hence higher desirability is obtained at high temperatures. Figure 15D showed that the immersion time showed a minimal effect on inhibition efficiency at high inhibitor concentrations but had a significant effect at lower concentrations. Based on the objective of achieving high inhibition efficiency over a longer period of immersion, high desirability is attained at a period of approximately 22 h (Figure 15C). The desirability also increased with increasing immersion time and temperature owing to the set objectives as shown in Figure 15E. Ultimately, Figure 15F shows that at higher temperatures, the immersion time effect on inhibition efficiency was greater than at lower temperatures. Triplicate gravimetric tests were conducted under these optimum conditions to validate the model. The average relative error was obtained by comparing the theoretical inhibition efficiency value with the experimental values according to Eq. 13. The average relative error obtained was 2.76%, from theoretical, IE values of 90.8, 90.6% and 85.9%. This confirmed the developed model’s suitability within the specified range of process parameters.

$$\begin{array}{c} Relative\;error\;\left(\%\right)=\frac{\left|experimental\;value-theoretial\;value\right|}{theoretial\;value}\times 100\% \end{array}$$ (13)

**TABLE 5:** Optimization constraints.

Name	Goal	Lower limit	Upper limit	Lower weight	Upper weight	Importance
A: Concentration	minimize	100	500	1	1	3
B: Temperature	is in range	25	65	1	1	3
C: Time	maximize	4	24	1	1	3
Inhibition Efficiency	maximize	78.5	93.3	1	1	3

### 3.5 Characterization

#### 3.5.1 Solubility of extracts

Both the okra-derived extracts were sparingly soluble in water and 1M HCl, to form a cream slimy solution. The extracts were practically insoluble in organic solvents such as ethanol, acetone, and chloroform. The solubility was also highly dependent on the concentration of the extract in the solution.

#### 3.5.2 FTIR analysis

**FIGURE 16:**
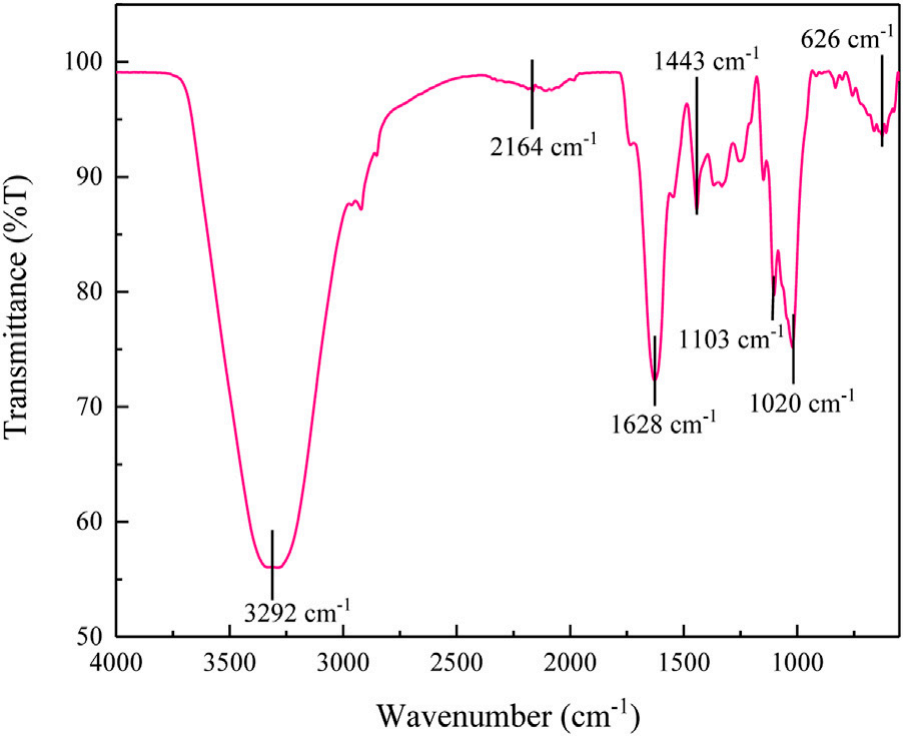
FTIR spectra of grafted okra mucilage.

**FIGURE 17:**
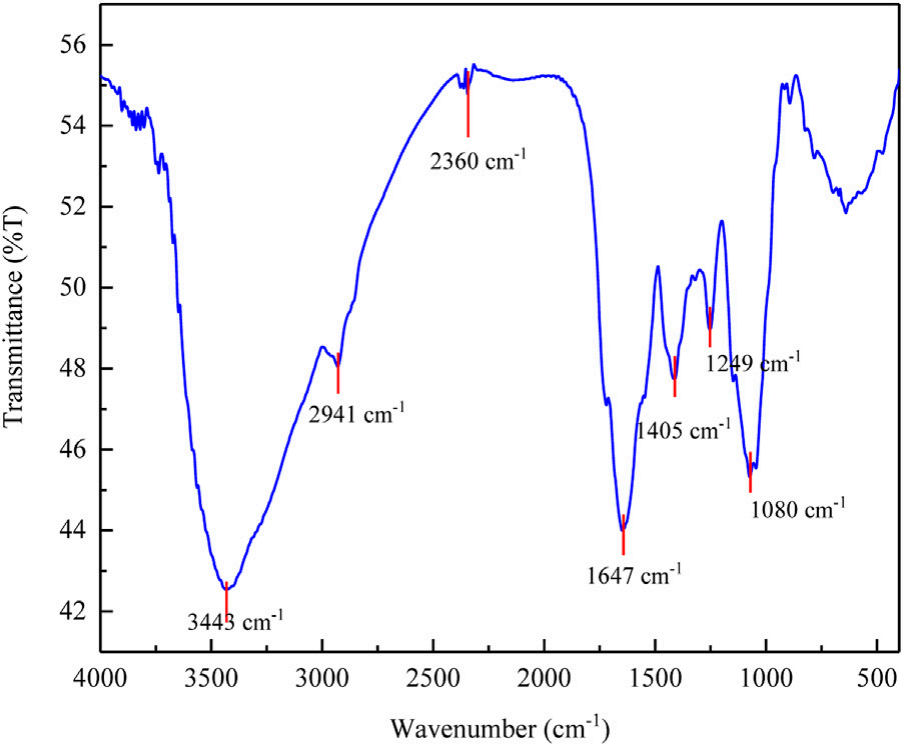
FTIR spectra of okra mucilage powder extract.

The FTIR spectrum of polyacrylamide-okra graft is shown in Figure 16. There is a visible difference in the spectrum of the copolymer compared to natural okra mucilage (Figure 17). The spectrum includes a characteristic peak at 1711.59 cm^-1^ representing C=O group, -NH bending at 1645.7 cm^-1^, -CN stretching at 1386.56 cm^-1^, and a -C-C-N asymmetric band at 1252.35 cm^-1^. These are all sign of grafting with acrylamide. The bands at 3190.38 cm^-1^ represent -OH stretching, while those at 2601.83 cm^-1^, and 2932.14 cm^-1^ may be assigned to aliphatic C-H bonds respectively. The medium peak at 1413.04 cm^-1^ is due to symmetric stretching of the -COO- group, while that at 1498.11 cm^-1^ represents the extending of C=C benzenoid rings. Similarly, the band at 881.34 cm^-1^ represents C-H out-of-plane bending (Geethanjali et al., 2014; Babaladimath et al., 2018). After 2801.83 cm^-1^ to about 1800 cm^-1^, broadening of the band is observed. In comparison to natural okra mucilage, this is attributed to overlapping between -NH of amide and -OH of mucilage, and an increase in hydrogen bonding in the amide groups (Mishra et al., 2008). Therefore, the results of FTIR analysis confirms the graft copolymerization of okra mucilage with acrylamide to form a graft copolymer of okra. Table 6 provides a summary of the FTIR investigations.

**TABLE 6:** Summary of the FTIR data for polyacrylamide-okra graft.

Wavenumber (cm^-1^)	Functional group
Broadband (1800–2801)	Overlapping between -NH of amide and -OH of mucilage
3190.4	-OH stretching
1711.6	C=O group
1645.7	-NH bending at
2602 and 2932	Aliphatic C-H stretching
1498.1	C=C stretching of benzenoid
1413	Symmetric stretching of the -COO-
1386.6	-CN stretching
1252.4	-C-C-N asymmetric band
Fingerprint region	Polysaccharides

#### 3.5.3 Thermogravimetric analysis

**FIGURE 18:**
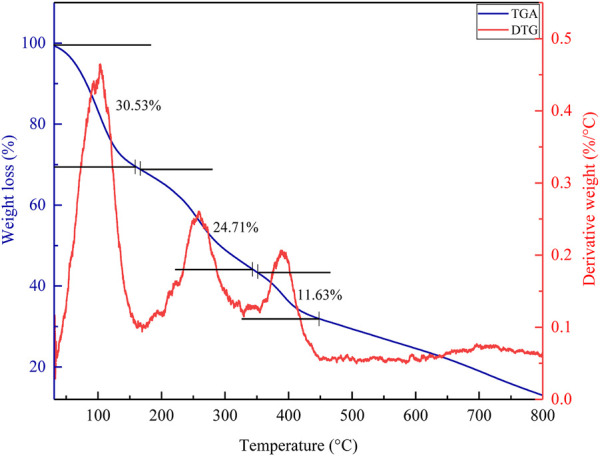
TGA analysis of grafted okra mucilage.

The decomposition pattern of the polyacrylamide-okra graft (Figure 18) was analysed with a change in temperature with heating performed up to a rate of 900°C at a rate of 10°C/min as depicted. Three distinct weight loss steps are evident from the weight loss profile. The first weight step (30.5%) occurs from 25°C–180 °C and is due to moisture loss. The second step (24.7%) from 180°C–350°C, and is due to polymer chain degradation, through the release of trapped gaseous pyrolysis products (Geethanjali et al., 2014). The third step from 350°C–450 °C (11.6%) indicates the breakdown of the polymer backbone, leaving behind very little residual ash. This profile demonstrates that grafting of the okra mucilage powder forms a copolymer, which is stable at higher temperatures.

#### 3.5.4 Surface morphology

**FIGURE 19:**
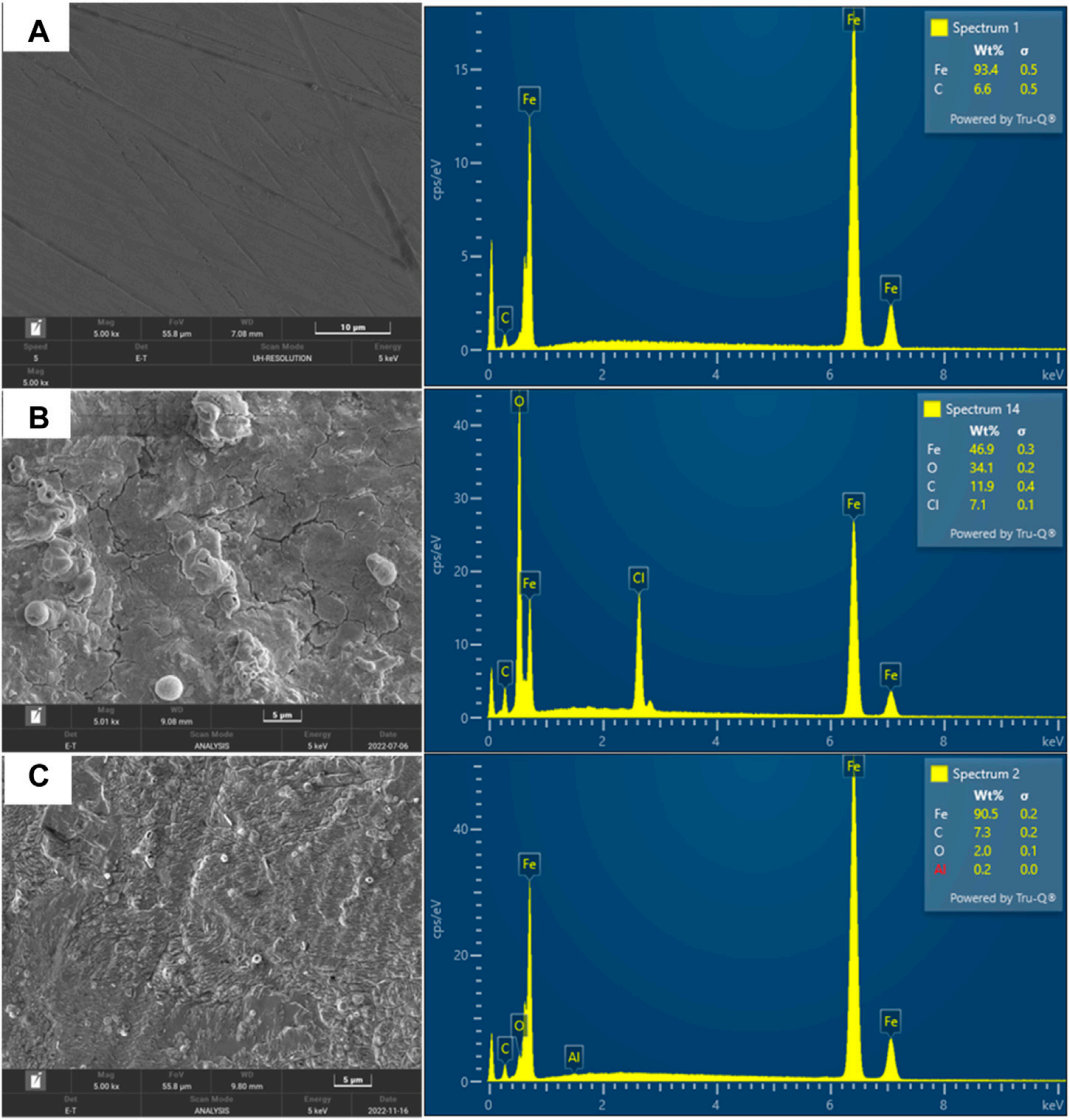
FESEM micrograph of mild steel metal: **(A)** before corrosion, **(B)** corrosion in the absence of inhibitor **(C)** corrosion in the presence of 800 ppm inhibitor.

FESEM analysis was used to examine the topology of the mild steel metal surface. Before and after 24 h of immersion in 1M HCl, FESEM micrographs of the polished MS surfaces were taken: before corrosion, immersion with no inhibitor, and immersion in the presence of 800 ppm of inhibitor. Figure 19A shows smooth surface of MS associated with polishing scratches, whereas the surface of the MS that is immersed with no inhibitor is associated with cavities and is heavily corroded as shown in Figure 19B. The inhibited sample shows the formation of an inhibitor film on the metal surface, with a less corroded surface as shown in Figure 19C. From the corresponding EDX spectra, the quantities of the Fe, O, and C weight percentages on the different samples was used to identify the extent of corrosion with representative EDX. The EDX graphs also show that the amount of O^2-^, which is a prerequisite for corrosion increases to 34.1 wt% on the MS surfaces when immersed with no inhibitor (Figure 19B) but decreases to 2 wt% in the presence of 800 ppm of inhibitor, resulting in obstruction from surface corrosion (Figure 19C). In Figure 19B, it is also seen that chloride ions (represented by Cl) and water molecules (represented by O) as corrosion products on the metal which was immersed without polyacrylamide-okra graft, which is evidence of corrosion products like iron hydroxide and iron chloride, which are absent in the inhibited solution (Mobtaker et al., 2021). It is also clear that in the uninhibited sample, the binding of chloride from the acid was higher than in the other samples. As shown in Figure 19C, the sample with polyacrylamide-okra graft inhibitor show the formation of a surface due to molecules adsorption.

## 4 Conclusion

RSM, in conjunction with three levels of BBD and gravimetric and electrochemical analyses, was successfully used to investigate and optimise the effect of concentration, temperature, and immersion time on the corrosion inhibition efficiency of polyacrylamide-okra graft. The polymer was characterized using FTIR and TGA methods. FTIR revealed a visible difference in the spectrum of the copolymer compared to natural okra mucilage with new functional groups such as -NH_2_ and -CN. Overlapping between -NH of amide and -OH of mucilage is also present which contributes to increased hydrogen bonding. TGA also showed that the polymer has high thermal stability. The inhibitor proved to efficiently inhibit corrosion for mild steel in 1M HCl and subscribed to Langmuir adsorption isotherm, physio-chemisorption mode of adsorption, and acted as a mixed-type inhibitor. The results from the quantitative analyses were in good agreement, where an increased inhibition effect was observed with an increase in inhibitor concentration from 20–800 ppm. The effect of factors on the corrosion inhibition of polyacrylamide-okra graft shows that temperature had the most predominant effect followed by the inhibitor concentration and then the immersion time. The quadratic model developed through ANOVA was used to optimize the inhibition process, where the,IE of 88.2% is achievable at 142.3 ppm, 60.4 °C, and 22.4 h. FESEM analysis proved that there was an inhibitor layer formed by adsorption to shield the metal surface from corrosion. Grafting natural okra appeared to be effective in increasing the performance of okra mucilage as a corrosion inhibitor at much lower concentrations, providing an efficient, low toxicity, cost-friendly, plant-based inhibitor alternative for enhancement and consideration towards mild steel corrosion in a 1M HCl.

## Data Availability

The original contributions presented in the study are included in the article/Supplementary Material, further inquiries can be directed to the corresponding author.
