# Supplementary material for: Quantitative and qualitative analyses of grafted okra for corrosion inhibition of mild steel in acidic medium
Source: Front Chem. 2023 Feb 24;11:1129673. doi: 10.3389/fchem.2023.1129673 (PMC9998522; doi:10.3389/fchem.2023.1129673)
Supplement: Supplementary file 1 [file Table1.pdf]

Table S1: Box-Benhken design matrix for three-factor-effect investigation and response value for polyacrylamide graft of okra

Std	Run	Factor 1	Factor 2	Factor 3	Response 1
		A: Concentration (ppm)	B: Temperature (°C)	C: Time (hrs)	IE (%)
5	1	100	45	4	83.5
12	2	300	65	24	89
~~7~~	~~3~~	~~100~~	~~45~~	~~24~~	~~12.9~~
11	4	300	25	24	83.9
14	5	300	45	14	91.3
6	6	500	45	4	86.5
~~8~~	~~7~~	~~500~~	~~45~~	~~24~~	~~36.5~~
13	8	300	45	14	90.1
4	9	500	65	14	93.3
10	10	300	65	4	88.9
15	11	300	45	14	92.2
16	12	300	45	14	91.2
3	13	100	65	14	90.3
17	14	300	45	14	91.1
9	15	300	25	4	80.1
1	16	100	25	14	78.5
2	17	500	25	14	83.5

Table S2: Summary of the model fitting of polyacrylamide graft of okra

Source	Sequential p-value	Lack of Fit p-value	Adjusted R^2	Predicted R^2	
Linear	**0.0079**	**0.0034**	**0.5488**	**0.3606**	**Suggested**
2FI	0.9421	0.0014	0.4075	-0.4445	
Quadratic	**0.0056**	**0.0218**	**0.9092**		**Suggested**
Cubic	0.0218		0.9738		Aliased
